# Spatial memory deficit across aging: current insights of the role of 5-HT_7_ receptors

**DOI:** 10.3389/fnbeh.2014.00448

**Published:** 2015-01-14

**Authors:** Gregory Beaudet, Valentine Bouet, Christelle Jozet-Alves, Pascale Schumann-Bard, François Dauphin, Eleni Paizanis, Michel Boulouard, Thomas Freret

**Affiliations:** Université de Caen Basse-Normandie, Groupe Mémoire et Plasticité Comportementale (GMPc), EA 4259Caen, France

**Keywords:** spatial cognition, Alzheimer’s disease, serotonin, aging, 5-HT_7_R

## Abstract

Elderly persons often face biological, psychological or social changes over time that may cause discomfort or morbidity. While some cognitive domains remain stable over time, others undergo a decline. Spatial navigation is a complex cognitive function essential for independence, safety and quality of life. While egocentric (body-centered) navigation is quite preserved during aging, allocentric (externally-centered) navigation—based on a cognitive map using distant landmarks—declines with age. Recent preclinical studies showed that serotonergic 5-HT_7_ receptors are localized in brain regions associated with allocentric spatial navigation processing. Behavioral assessments with pharmacological or genetic tools have confirmed the role of 5-HT_7_ receptors in allocentric navigation. Moreover, few data suggested a selective age-related decrease in the expression of 5-HT_7_ receptors in pivotal brain structures implicated in allocentric navigation such as the hippocampal CA3 region. We aim to provide a short overview of the potential role of 5-HT_7_ receptors in spatial navigation, and to argue for their interests as therapeutic targets against age-related cognitive decline.

The world’s population is aging at an unprecedented rate and constitutes a significant public health issue. Aging is associated with the decline in selective aspects of cognitive performance, together with brain functional and anatomical changes. Among cognitive functions that decline with age, spatial navigation is impaired after the age of 60, with an acceleration in decline after 70 (Barrash, 1994). Spatial navigation is a complex cognitive ability that is essential for independence, safety and quality of life. Spatial navigation capacities have also recently attracted attention in the field of neurodegenerative disorders, especially Alzheimer’s disease (AD; Lithfous et al., 2013). Indeed, from a clinical point of view, diagnosis of AD in very early stages appears to be a crucial challenge to optimize therapeutic management. The modulation of the last discovered serotonin receptors, 5-hydroxytryptamine receptors type 7 or 5-HT_7_R, might be a promising therapeutic approach.

## Egocentric vs. allocentric spatial strategy shift across aging

Spatial navigation refers to the process of determining and maintaining a course or trajectory to a goal location (Franz and Mallot, 2000). Two main kinds of strategies are commonly distinguished, depending on the frame of reference used to encode location: egocentric vs. allocentric (Zaehle et al., 2007). When using an egocentric frame of reference, spatial information is encoded from the viewpoint of the individual: it corresponds to self-centered navigation. For example, an egocentric strategy can refer to the association between a particular landmark and left or right body turn in response to this landmark (e.g., turn right at the bookshop, then turn left at the museum). Allocentric navigation is based on non-self-centered maps, i.e., independent of the individual position (O’Keefe and Nadel, 1978). The individual memorizes spatial relationships between landmarks, such as relative direction, angle and distances (Dolins and Mitchell, 2010). Unlike egocentric strategies, allocentric strategies enable an individual to plan novel routes during navigation.

Among the numerous experimental procedures designed to assess spatial learning abilities, most of them allow only one kind of strategy to be used. However, some paradigms offer the opportunity to use either an egocentric or an allocentric strategy (Paul et al., 2009). Several studies have shown that even though individuals spontaneously select one strategy, they are still able to switch when necessary (*humans*: Iaria et al., 2003; Etchamendy and Bohbot, 2007; Iglói et al., 2009; *rodents*: Gibson and Shettleworth, 2005). Thus, this coexistence of strategies allows individuals to adapt themselves to environmental constraints, by shifting for instance from one strategy to another when some spatial information becomes unreliable (Healy, 1998; Shettleworth, 2009).

Functional neuroimaging and neuropsychological studies have provided evidence that the hippocampus and para-hippocampal areas are critically involved when using an allocentric frame of reference (Bohbot et al., 1998; Maguire et al., 1998; Moffat et al., 2006). Conversely, right-sided parietal association cortices and subcortical regions, especially the striatum (i.e., caudate nucleus and putamen), are commonly associated with egocentric strategies (Maguire et al., 1998; Hartley et al., 2003; Iaria et al., 2003; Galati et al., 2010). Indeed, while performing a spatial task that offers the choice between the two strategies, the subjects who preferentially use an allocentric strategy display a high activity in the right hippocampus; whereas higher brain activity is observed in the caudate nucleus when the egocentric strategy was preferentially adopted (Iaria et al., 2003). Similar results have been observed in preclinical studies. Through the use of different experimental approaches (lesional, transient inactivation or pharmacological experiments), animal data suggested that egocentric and allocentric strategies rely on different neural networks in rodents: the dorso-striatal and the hippocampal mnesic systems, respectively (Packard and McGaugh, 1992; Packard, 1999; DeCoteau and Kesner, 2000; Miranda et al., 2006; Burgess, 2008). When performing a behavioral task in which both strategies are efficient, a higher neuronal activity (assessed through phospho-CREB immunoreactivity) is observed in the dentate gyrus, hippocampal CA1 and CA3 of rats preferentially using an allocentric strategy. Conversely, dorso-lateral and dorso-medial striatum are more activated in rats preferentially using an egocentric strategy. This parallel functioning is however not always straightforward, since both strategies rely on, at least, some shared brain structures. In humans, the posterior parietal and the frontal cortex appear to be activated both in individuals preferring an egocentric or an allocentric strategy (Iaria et al., 2003). In rodents, although the CA3 is activated when using an allocentric strategy, and conversely the striatum when using an egocentric strategy, the dentate gyrus and mammillary bodies are activated in both cases (Rubio et al., 2012; see Table 1).

**Table 1:** **Relative abundance of 5-HT_7_R within brain structures involved in egocentric and/or allocentric spatial strategies, and evolution of 5-HT_7_R mRNA expression across aging**.

Brain structures	5-HT_7_ receptors		mRNA expression across Aging
	Egocentric	Allocentric	
Dorsal striatum	**XX**		NI
Thalamus (anterior nucleus)	X	X	≈
Hypothalamus (mammillary nucleus)	X	X	≈
Amygdala	X		≈
Cerebellum		X	NI
**Hippocampal formation**			
CA1	X	X	≈
CA3	X	**XX**	↘
DG	X	X	≈
**Cortex**			
Medial prefrontal	X	X	NI
Frontal		X	NI
Temporal		X	NI
Parietal	X	X	NI
Occipital		X	NI

**Relative density of 5-HT_7_R**:

few

medium

many

*Abundance of 5-HT_7_R are displayed in gray-scale. “X” indicates an involvement in navigational strategies, “XX” highlights the main structure involved in either egocentric or allocentric strategy. “NI” = Non Investigated, and “↘”= decrease of 5-HT_7_R mRNA expression, “≈” = no change. Table has been drawn according to the following publications in animals: (To et al., 1995; Gustafson et al., 1996; Duncan et al., 1999; Yau et al., 1999; Kohen et al., 2000; Begega et al., 2001; Neumaier et al., 2001; Moffat and Resnick, 2002; Colombo et al., 2003; Bonaventure et al., 2004; Varnäs et al., 2004; Moffat et al., 2006; Duncan and Franklin, 2007; Begega et al., 2012; Rubio et al., 2012)*.

**Table 1:**
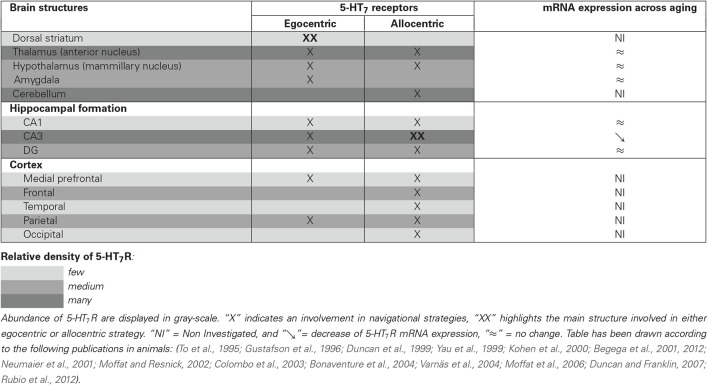


Advanced age is known to alter spatial cognitive abilities (Moffat, 2009; Klencklen et al., 2012). Thus, healthy elders have poorer performances than their younger counterparts when learning a route (i.e., egocentric strategy) (Moffat et al., 2001; Wiener et al., 2013). Depending on the procedure used, this impairment is not always observed (Etchamendy et al., 2012; Gazova et al., 2013), or sometimes remains quite moderate (Pouliot and Gagnon, 2005). However, when addressing allocentric navigation (using either real or virtual environments), age-related impairments are more systematically observed (Moffat and Resnick, 2002, 2007; Driscoll et al., 2003, 2005; Iaria et al., 2009; Gazova et al., 2013). In addition, compared to children, the proportion of subjects preferentially choosing an allocentric strategy in a spatial task is decreased in elders (Bohbot et al., 2012; Rodgers et al., 2012). Aging seems to specifically impair the ability of switching to an allocentric strategy, when an egocentric strategy becomes unreliable (Harris et al., 2012). All results above are consistent with animal studies, showing age-related impairments when using an allocentric strategy (Gallagher and Pelleymounter, 1988; Begega et al., 2001). Additionally, even if aged mice are still able to use an allocentric strategy, they preferentially use an egocentric strategy when they have the opportunity to choose (Nicolle et al., 2003). According to Klencklen et al., impairments observed in egocentric tasks may be due to deficits in the planning of the pathway, while deficits observed in allocentric tasks may be due to the lower likelihood of elderly people to learn configurational information about the environment (Klencklen et al., 2012).

Several studies have been performed to better understand the neural mechanisms underlying age-related alteration in spatial abilities. From a neuroanatomical point-of-view regarding age-induced changes, a shrinkage of the caudate nucleus as well as a greatest deterioration of the frontal cortex are among the two modifications the most frequently documented and well-admitted (Raz et al., 1997, 2003; Greenberg et al., 2008; Kalpouzos et al., 2009; Walhovd et al., 2011). In regards to the hippocampus, while most studies found a shrinkage with aging (Du et al., 2006), some did not found any (Good et al., 2001). This discrepancy may be due to the sampling method used: e.g., studies undertaken across a wide spread of ages (e.g., from 18 to 79 in Good et al., 2001) vs. key periods of age-related impairments (e.g., from 58 to 87 years old in Du et al., 2006; Kalpouzos et al., 2009). Beyond these differences, it seems that shrinkage is usually observed across aging only when considering the posterior part of the hippocampus. Hippocampal neuronal integrity has also been shown to be altered in elderly subjects (Driscoll et al., 2003), and a reduced resting-state metabolism has been observed (Small et al., 2002). Moreover, a reduced activation of the neural network underlying allocentric strategy was observed in older adults (Moffat et al., 2006). Konishi and Bohbot showed that the volume of gray matter in the hippocampus positively correlates with spontaneous allocentric strategies in the healthy elderly (Konishi and Bohbot, 2013). Structural, neurochemical and functional age-related changes in hippocampus and associated structures may explain why impairments are observed in allocentric strategies in the elderly.

## 5-HT_7_ receptors and spatial navigation

The 5-HT_7_R belong to the superfamily of G-protein-coupled receptors. Since their discovery, almost 20 years ago, 5-HT_7_R distribution has been accurately investigated both in humans and in non-human species (*human*: Varnäs et al., 2004; *non-human*: To et al., 1995; Waeber and Moskowitz, 1995; Gustafson et al., 1996; Neumaier et al., 2001; Bonaventure et al., 2004). Even though slight differences can emerge according to the protocols or the techniques used—immunohistochemistry, radiolabelling, qPCR, etc…, the central distribution of 5-HT_7_R is generally in accordance within the three mainly studied species (human, rat and guinea pig) (Leopoldo et al., 2011). In the central nervous system, a higher density of 5-HT_7_R was found in the hippocampus (particularly in the CA3 and the dentate gyrus, and to a lesser extent in the CA1), in the hypothalamus and in the thalamus (particularly within the anterior part). Additionally, 5-HT_7_R are also broadly—but less densely—distributed in the cortex, especially in the frontal, piriform and cingulate cortices, as well as in the amygdala and the dorsal raphe nucleus. As regards to its expression in basal ganglia nuclei, results show discrepancies across species. In humans, a high concentration is observed in caudate and putamen nuclei, whereas the striatum appears to be a structure with low 5-HT_7_R expression in rats (Martín-Cora and Pazos, 2004).

Given their distribution within the central nervous system, several recent experiments support a role for 5-HT_7_R in learning and memory processes (for review see Meneses, 2013). Most interestingly, it seems that 5-HT_7_R plays a major role in hippocampus-dependent memory processing (Roberts et al., 2004; Gasbarri et al., 2008; Sarkisyan and Hedlund, 2009), particularly when learning a location is required to solve the task This has notably been observed while using two different paradigms of the object recognition test. Typically, the test consisted in two consecutive sessions separated by an intersession interval, and is carried out in an open-field. During the first session, animal can freely explore two similar objects. During the second session, according to the paradigm used, one of the two objects is either replaced by a new one (this paradigm is called novel object recognition test), or only displaced (this paradigm is called object-place recognition test (Oliveira et al., 2010)). The difference between those two paradigms of recognition task relies on the more pronounced involvement of the hippocampus in the object-place recognition test (Barker and Warburton, 2011). In the “novel” object recognition test, transgenic 5-HT_7_R knock-out (KO) mice performed similarly to their wild-type siblings (Sarkisyan and Hedlund, 2009). As a contrary, in the object-place recognition test, while the spatial component of the test is more challenging, KO mice displayed a marked impairment of memory performances. Of note, the same hold true if wild mice are administered before the first session, with SB-269970, a selective 5-HT_7_R antagonist. Indeed, whereas no differences are noticed for the non-spatial paradigm of the test (novel object recognition) when the antagonist is administrated, impairments are observed in the spatial version of the recognition task (Sarkisyan and Hedlund, 2009). Thus, those two results obtained with either KO mice or after administration of 5-HT_7_R antagonist both argue for a predominant role of 5-HT_7_R in behavioral tasks requiring animals to learn location of objects in an allocentric frame of reference. Still in the context of pharmacological modulation of 5-HT_7_R, a sub-chronic treatment during adolescence withLP-211 (a selective 5-HT_7_R agonist) did not lead to major modifications of performances in the novel object recognition paradigm at adulthood (Canese et al., 2014). Unfortunately, the effect of an acute tonic pharmacological modulation of 5-HT_7_R has yet not been investigated in the two paradigms of the recognition test. Again, such a result is in favor of an involvement of 5-HT_7_R in memory processes when spatial learning is required.

Beyond the importance of the spatial component in the behavioral task, the involvement of 5-HT_7_R could also vary according to the strategy used to perform the test. In fact, KO mice have been tested in the Barnes maze test (Roberts et al., 2004; Sarkisyan and Hedlund, 2009). This dry-land maze consists of a brightly illuminated elevated circular platform (aversive stimulation), with several holes around its perimeter (Barnes, 1979). Each session of the test starts by placing the animal in the middle of the maze. Animals will then try to escape the aversive bright light maze by searching and entering into the box placed underneath one of the holes. Even though egocentric and allocentric strategies are both efficient in this test (Harrison et al., 2006), a recent study has demonstrated that all of the 13 inbred strains of mice tested preferentially used an egocentric strategy to find the platform (O’Leary et al., 2011). In this task, KO mice did no display any spatial memory impairment (Roberts et al., 2004; Sarkisyan and Hedlund, 2009). Thus, this lack of deficit might be explained by the use of a striatum-dependent egocentric strategy, spared in these KO mice (Sarkisyan and Hedlund, 2009). Besides, it has been elegantly demonstrated that KO mice displayed a clear deficit in allocentric strategy. Indeed, Sarkisyan and Hedlund performed a reversal test a month later during which the location of the escape box was moved 180° away from its original location. As expected, all mice started looking for the escape box at its original quadrant. But, after having explored the now empty hole, the two populations of mice (KO and wild-type) started to explore the maze in a quite different manner. Indeed, while KO mice found themselves close to the starting position, they continued to use the initial pathway to come back to the empty escape box. Such observation led the authors to hypothesize that KO mice may not be able to switch to an allocentric strategy, so keep using an egocentric one, even though it is no longer reliable. Such behavioral impairments observed in KO mice might be related to the absence of expression of 5-HT_7_R in the CA3 region of the hippocampus and dentate gyrus. Indeed, those two regions are mainly involved in the integration of environmental changes. Besides, in wild type mice, a high density of 5-HT_7_R is observed in the CA3 region of the hippocampus and dentate gyrus, relative to other brain regions. Thus, the lack of expression of 5-HT_7_R within those two brain regions in KO mice would have led to an impairment of allocentric strategy. Unfortunately, the authors did not test this hypothesis with another group of wild-type mice within the same experimental paradigm, but with the presence of a wall around the edge of the maze. Such a group is considered as a “negative control”. Indeed, in such condition, since no external visual cue is available, animals are forced to use an egocentric strategy at every step of the test.

Finally, the group of Meneses also investigated the involvement of 5-HT_7_R in another spatial task, namely the radial arm maze (Gasbarri et al., 2008). In this work, authors observed conflicting results to what we previously described. Indeed, they showed that the pharmacological blockade of 5-HT_7_R (SB-269970) improved spatial learning performances. Thus, the same pharmacological agent (namely SB-269970) has been demonstrated both to improve (Gasbarri et al., 2008) (see above) or to impair spatial memory performances (Sarkisyan and Hedlund, 2009). Roberts and Hedlund have suggested an explanation that allows for overcoming discrepancies (Roberts and Hedlund, 2012). In fact, in the radial arm maze experiment, the animal is always placed at the center of the maze at the beginning of each session. Thus, they suggested that learning the location of the baited-arm through an egocentric strategy (a direct route from the starting position) could be more efficient than the allocentric one. If this is true, then the better performances observed in treated rats may be explained by the fact that SB-269970 would have favored a more rigid strategy (independent of 5-HT_7_R).

## 5-HT_7_R across aging: insight to a potential therapeutic target?

Surprisingly, only a few studies have examined changes in brain 5-HT_7_R density during aging, with only five publications from three different research groups available so far. Unfortunately, the results are not straightforward. The first publication comes from the Seckl lab which used a pharmacological model of adrenalectomy in order to mimic accelerated aging in rats (Yau et al., 1997). In this work, the authors observed, through *in situ* hybridization, an increased expression of 5-HT_7_R mRNA expression in the hippocampal CA3 regions 24 h after the induction of the experimental model (Yau et al., 1997). However, when 2 years later the same group performed the same experiments but in aged rats (22–24 months), no modification was observed in any of the hippocampal sub-regions (Yau et al., 1999). This apparent discrepancy seems thus more related on the relevance, in the context of 5-HT_7_R, of the pharmacological model used in the first study in order to mimic aging processes. Kohen et al. have also explored changes in 5-HT_7_R density across aging in three groups of rats, aged either 3, 12 or 24 months, modeling different stages of aging; adult, middle-aged and old, respectively (Kohen et al., 2000). In this work, the authors have studied the subfields of the hippocampus by separating the ventral and dorsal part. In accordance to the work of Yau et al. in aged rats (Yau et al., 1999), the authors did not observe any change in the dorsal CA3 of the hippocampus. However, they noticed an age-related decline in the ventral CA3 part of the hippocampus. This decrease, already marked in the middle-aged group of rats, remained the same in old rats (24 months). Finally, using another animal model (hamsters), Duncan and Franklin also investigated the effect of aging on mRNA expression of 5-HT_7_R in discrete forebrain and midbrain regions (Duncan and Franklin, 2007). Three age groups of hamsters (3–5, 12–14 and 17–19 months of age) were also studied. Contrary to what was previously observed, this work has not highlighted alteration of mRNA expression across aging in any of the investigated brain regions—including the hippocampus, with the exception of the decrease measured in the cingulate cortex and the paraventricular thalamic nucleus. In a previous study which focused in four brain structures that regulate circadian cycle (namely the suprachiamastic nuclei, the lateral geniculated nuclei, the median raphe nucleus and dorsal raphe nucleus), the same group of researchers also observed a marked decrease in 5-HT_7_R in the dorsal raphe nucleus (Duncan et al., 1999). As regards to expression of 5-HT_7_R mRNA in the hippocampus, several explanations can be proposed to explain discrepancies observed in the effects of aging. Indeed, the different animal models used (rats, mice, hamsters) may account for such divergent results, as well as, for instance, the location of the brain slice performed in the different works (highlighting or masking discrete regions of interest), etc… Besides, one may note that when comparing the works of the group of Yau and Kohen, if the whole structure is taken into account, no age-induced modification is observed, whereas a marked decreased of approximately 30% is well observed when focusing in the ventral part of the hippocampus. Considering the role of the CA3 region of the hippocampus in spatial strategy highlighted above, a decreased expression of 5-HT_7_R in this brain structure could account for impairments of the shift between spatial strategies across aging.

## Conclusion and future directions

Regarding the question of aging-induced deficit in the shift between egocentric and allocentric strategies and the role of 5-HT_7_R in these impairments, a crucial lack of data makes it difficult to draw conclusions presently. Indeed, all the previously cited studies exploring the changes of 5-HT_7_R across aging have occurred at the messenger RNA level; but not at the level of the final product, i.e., the protein. Indeed, changes in post-transcriptional and/or translational regulation mechanisms may lead to divergent observations between those two levels. In fact, this has already been observed in the context of 5-HT_7_R. Thus, in 2007, Duncan and Franklin have not shown any age-induced modification of 5-HT_7_R mRNA expression in the dorsal raphe nuclei (Duncan and Franklin, 2007). However, the same group of researchers observed a marked decrease of 50% at the protein level of 5-HT_7_R in dorsal raphe nuclei in a prior study (Duncan et al., 1999). Thus, changes in the level of transcription of the 5-HT_7_R mRNA did not account for the age-related difference observed at the protein level, at least in this brain structure. Besides, 5-HT_7_R might also be putatively subjected, across aging, to modifications in their affinity or to changes in their coupling to G-proteins or other signaling pathways. In fact, such an effect has already been suggested by Duncan et al. (2004). Thus, even though a lack of data prevents us from drawing strong conclusions about the role of 5-HT_7_R in the shift from spatial strategies across aging, those receptors appear to be promising target given their known locations in cerebral structures affected by aging processes, and their demonstrated role in spatial paradigms requiring the use of an allocentric strategy.

## Conflict of interest statement

The authors declare that the research was conducted in the absence of any commercial or financial relationships that could be construed as a potential conflict of interest.
